# Supplementary material for: Investigating the effects of incremental conditioning and supplemental dietary tryptophan on the voluntary activity and behaviour of mid-distance training sled dogs
Source: PLoS One. 2020 Aug 13;15(8):e0232643. doi: 10.1371/journal.pone.0232643 (PMC7425858; doi:10.1371/journal.pone.0232643)
Supplement: S1 Table — 1Distance ran on days when behavioural observations took place. 2Standard error of the mean; n = 10 for wks 0 to 2, 4 to 6 and 8 to 11; n = 9 for wk 7; n = 8 for wk 3. (DOCX) [file pone.0232643.s001.docx]

|  | **Week (Distance ran^1^)** | | | | | | | | | | | |  | **p-value** |  |
| --- | --- | --- | --- | --- | --- | --- | --- | --- | --- | --- | --- | --- | --- | --- | --- |
|  | **0** | **1** | **2** | **3** | **4** | **5** | **6** | **7** | **8** | **9** | **10** | **11** |  |  |  |
| **Behaviour** | **(8.9 km)** | **(12.9 km)** | **(19.7 km)** | **(26.7 km)** | **(30.8 km)** | **(38.4 km)** | **(30.2 km)** | **(30 km)** | **(30 km)** | **(53.2 km)** | **(30.2 km)** | **(38.2 km)** | **SEM^2^** | **Treatment** | **Week* Treatment** |
| Chewing |  |  |  |  |  |  |  |  |  |  |  |  |  |  |  |
| Control | 0 | 0 | 0 | 0 | 0 | 0.7 | 0 | 0 | 0 | 0 | 0 | 0 | 0.2 | 0.807 | 0.714 |
| Treatment | 0 | 0.1 | 0.3 | 0 | 0 | 0.1 | 0 | 0 | 0 | 0 | 0 | 0 | 0.2 |  |  |
| Digging |  |  |  |  |  |  |  |  |  |  |  |  |  |  |  |
| Control | 0.2 | 1.1 | 0.3 | 1.1 | 1.6 | 0.2 | 4.6 | 0 | 0.3 | 0 | 0 | 0 | 1.2 | 0.364 | 0.501 |
| Treatment | 0 | 0.1 | 0.5 | 0.2 | 1.0 | 0.0 | 0 | 0 | 0 | 0 | 1.5 | 0 | 1.2 |  |  |
| Jumping |  |  |  |  |  |  |  |  |  |  |  |  |  |  |  |
| Control | 0.2 | 1.6 | 0.8 | 0.6 | 0.6 | 1.7 | 1.4 | 0 | 0.3 | 0 | 0.3 | 0 | 1.3 | 0.545 | 0.254 |
| Treatment | 1.8 | 1.7 | 2.9 | 2.4 | 2.8 | 0.5 | 0.6 | 0 | 3.3 | 0 | 0.7 | 0.6 | 1.3 |  |  |
| Lunging |  |  |  |  |  |  |  |  |  |  |  |  |  |  |  |
| Control | 4.0 | 10.2 | 6.7 | 4.8 | 4.9 | 3.4 | 4.7 | 0 | 3.7 | 0 | 0.7 | 0 | 3.8 | 0.888 | 0.986 |
| Treatment | 0.9 | 7.9 | 8.6 | 8.4 | 7.6 | 3.8 | 1.4 | 0 | 5.1 | 0 | 3.2 | 1.9 | 3.8 |  |  |
| Postural Changes |  |  |  |  |  |  |  |  |  |  |  |  |  |  |  |
| Control | 26.7 | 35.1 | 31.9 | 24.9 | 22.7 | 26.5 | 25.5 | 19.9 | 26.3 | 4.2 | 7.3 | 6.6 | 10.8 | 0.413 | 0.692 |
| Treatment | 37.2 | 53.4 | 51.9 | 40.6 | 46.1 | 38.3 | 25.0 | 23.3 | 25.6 | 1.4 | 20.1 | 18.0 | 10.8 |  |  |
| Sitting |  |  |  |  |  |  |  |  |  |  |  |  |  |  |  |
| Control | 24.9 | 1.3 | 19.6 | 9.3 | 2.0 | 2.4 | 4.3 | 5.1 | 5.3 | 21.7 | 13.5 | 15.2 | 10 | 0.652 | 0.989 |
| Treatment | 20.1 | 2.1 | 1.9 | 3.3 | 1.4 | 0.9 | 6.7 | 0 | 4.9 | 19.6 | 20.8 | 8.5 | 10 |  |  |
| Standing |  |  |  |  |  |  |  |  |  |  |  |  |  |  |  |
| Control | 40.1 | 46.4 | 38.9 | 46.7 | 62.9 | 63.4 | 58.2 | 54.4 | 59.5 | 29.1 | 71.7 | 70.6 | 14.9 | 0.372 | 0.765 |
| Treatment | 38.5 | 32.7 | 33.2 | 42.1 | 39.2 | 56.4 | 68.4 | 56.9 | 53.6 | 29.9 | 34.5 | 40.3 | 14.9 |  |  |
| Lying |  |  |  |  |  |  |  |  |  |  |  |  |  |  |  |
| Control | 3.7 | 3.6 | 0 | 7.8 | 4.7 | 1.6 | 0 | 19.3 | 0.1 | 44.9 | 6.4 | 7.5 | 9.6 | 0.684 | 0.891 |
| Treatment | 0 | 0 | 0 | 0.6 | 0 | 0 | 0 | 19.4 | 7.5 | 49.1 | 19.1 | 30.5 | 9.6 |  |  |
