# Supplementary material for: Investigating the effects of incremental conditioning and supplemental dietary tryptophan on the voluntary activity and behaviour of mid-distance training sled dogs
Source: PLoS One. 2020 Aug 13;15(8):e0232643. doi: 10.1371/journal.pone.0232643 (PMC7425858; doi:10.1371/journal.pone.0232643)
Supplement: S2 Table — 1Distance ran on days when behavioural observations took place. 2Standard error of the mean; n = 10 for wks 0 to 2, 4 to 6 and 8 to 11; n = 9 for wk 7; n = 8 for wk 3. (DOCX) [file pone.0232643.s002.docx]

|  | **Week (Distance ran^1^)** | | | | | | | | | | | |  | **p-value** |  |
| --- | --- | --- | --- | --- | --- | --- | --- | --- | --- | --- | --- | --- | --- | --- | --- |
|  | **0** | **1** | **2** | **3** | **4** | **5** | **6** | **7** | **8** | **9** | **10** | **11** |  |  |  |
| **Behaviour** | **(8.9 km)** | **(12.9 km)** | **(19.7 km)** | **(26.7 km)** | **(30.8 km)** | **(38.4 km)** | **(30.2 km)** | **(30 km)** | **(30 km)** | **(53.2 km)** | **(30.2 km)** | **(38.2 km)** | **SEM^2^** | **Treatment** | **Week*Treatment** |
| Sitting |  |  |  |  |  |  |  |  |  |  |  |  |  |  |  |
| Control | 5 | 5.1 | 12.4 | 14.4 | 2.5 | 9.3 | 6.2 | 5.5 | 3.0 | 13.4 | 4.9 | 9.5 | 11.5 | 0.503 | 0.925 |
| Treatment | 18.7 | 16.5 | 16.6 | 6.0 | 5.8 | 18.5 | 7.1 | 1.4 | 23.0 | 26.7 | 20.7 | 14.5 | 11.5 |  |  |
| Standing |  |  |  |  |  |  |  |  |  |  |  |  |  |  |  |
| Control | 69.3 | 74.6 | 67.5 | 81.5 | 56.2 | 67.6 | 91.1 | 74.3 | 43.5 | 75.6 | 68.6 | 40.9 | 15.9 | 0.858 | 0.463 |
| Treatment | 81.3 | 74.3 | 74.2 | 94.6 | 83.9 | 60.4 | 91.0 | 78.2 | 27.1 | 43.5 | 42.2 | 30.8 | 15.9 |  |  |
| Lying |  |  |  |  |  |  |  |  |  |  |  |  |  |  |  |
| Control | 22.9 | 19.9 | 18.7 | 3.9 | 39.9 | 28.7 | 1.3 | 18.7 | 52.1 | 16.5 | 26.1 | 38.2 | 14.5 | 0.738 | 0.837 |
| Treatment | 2.8 | 10.5 | 10.5 | 0.9 | 9.9 | 22.5 | 3.2 | 21.6 | 51.3 | 30.4 | 38.5 | 55.8 | 14.5 |  |  |
